# A novel fluorescent probe for imaging the process of HOCl oxidation and Cys/Hcy reduction in living cells†

**DOI:** 10.1039/c7ra13419c

**Published:** 2018-03-05

**Authors:** Zhen Liu, Guoping Li, Yana Wang, Jiulong Li, Yang Mi, Linna Guo, Wenjian Xu, Dapeng Zou, Tiesheng Li, Yangjie Wu

**Affiliations:** The College of Chemistry and Molecular Engineering, Zhengzhou University Zhengzhou 450001 Henan Province P. R. China liguoping@zzu.edu.cn; School of Basic Medical Sciences, Zhengzhou University Zhengzhou 450001 Henan Province P. R. China

## Abstract

A new on-off-on fluorescent probe, CMOS, based on coumarin was developed to detect the process of hypochlorous acid (HOCl) oxidative stress and cysteine/homocysteine (Cys/Hcy) reduction. The probe exhibited a fast response, good sensitivity and selectivity. Moreover, it was applied for monitoring the redox process in living cells.

## Abstract 2

A new on–off–on fluorescent probe, CMOS, was designed and applied to detect the process of HOCl oxidation and Cys/Hcy reduction.

## Graphical abstract

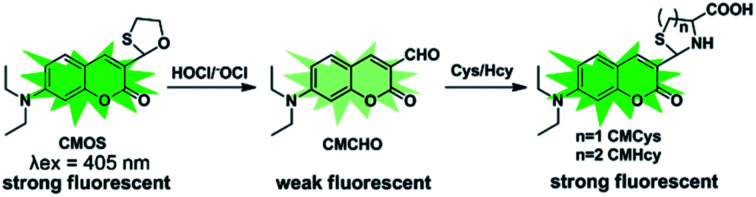

Reactive oxygen species (ROS) are indispensable products and are closely connected to various physiological processes and diseases.^1^ For instance, endogenous hypochlorous acid (HOCl) as one of the most important ROS, which is mainly produced from the reaction of hydrogen peroxide with chloride catalyzed by myeloperoxidase (MPO), is a potent weapon against invading pathogens of the immune system.^2,3^ However, excess production of HOCl may also give rise to oxidative damage *via* oxidizing or chlorinating the biomolecules.^4^ The imbalance of cellular homostasis will cause a serious pathogenic mechanism in numerous diseases, including neurodegenerative disorders,^5^ renal diseases,^6^ cardiovascular disease,^7^ and even cancer.^8^ Fortunately, cells possess an elaborate antioxidant defense system to cope with the oxidative stress.^9^ Therefore, it is necessary and urgent to study the redox process between ROS and antioxidants biosystems.

Fluorescence imaging has been regarded as a powerful visual methodology for researching various biological components as its advantages of high sensitivity, good selectivity, little invasiveness and real-time detection.^10,11^ To date, amounts of small molecular fluorescent probes have been reported for detection and visualization of HOCl *in vivo* and *in vitro*.^12–22,29^ The designed strategies of HOCl sensitive probes are based on various HOCl-reactive functional groups, such as *p*-methoxyphenol,^13^*p*-alkoxyaniline,^14^ dibenzoyl-hydrazine,^15^ selenide,^16^ thioether,^17^ oxime,^18^ hydrazide,^19^ hydrazone.^20^ But, many of these probes display a delayed response time and low sensitivity. And, only few fluorescent probes can be applied for investigating the changes of intracellular redox status.^21^ Besides, it's worth noting that most of the redox fluorescent probes rely on the organoselenium compounds.^22^ Even though these probes are well applied for detection of cellular redox changes, excessive organic selenium is harmful to organisms and the synthesis of organoselenium compounds is high requirement and costly. Additionally, almost all the reports have only investigated the reduction effects of glutathione (GSH) as an antioxidant in the redox events. While, there are the other two important biothiols, cysteine (Cys) and homocysteine (Hcy), which not only present vital antioxidants, but also are tightly related to a wide variety of pathological effects in biosystem, such as slowed growth, liver damage, skin lesions,^23^ cardiovascular,^24^ and Alzheimer's diseases.^25^ However, the fluorescent probes for specially studying internal redox changes between HOCl and Cys/Hcy are rarely reported. In this respect, a novel redox-responsive fluorescent probe, CMOS, was designed and synthesized in this work, and we hope that it can be a potential tool for studying their biological relevance in living cells.

Based on literature research, the aldehyde group has excellent selectivity in identification of Cys/Hcy, and the thiol atom in methionine can be easily oxidized to sulfoxide and sulfone by HOCl.^26,27^ Considering these two points, we utilized 2-mercaptoethanol to protect the 3-aldehyde of 7-diethylamino-coumarin as the recognition part of HOCl, meaning that two kinds of potential recognition moieties are merged into one site. Fluorescent probe CMOS can be easily synthesized by the acetal reaction in one step (Scheme S1†). A control molecule CMOS-2 was also prepared by 3-acetyl-7-diethylaminocoumarin (CMAC) similarly. The structure of all these compounds have been convinced by ^1^H NMR, ^13^C NMR, and HR-MS (see ESI†).

As shown in Scheme 1a, we estimated that both CMOS and CMOS-2 can be rapidly oxidized in the appearance of HOCl. The oxidation product CMCHO of CMOS, which has the aldehyde moiety, can further react with Cys/Hcy to obtain the final product CMCys and CMHcy, respectively. In contrast, the oxidation product CMAC of CMOS-2 cannot combine with Cys/Hcy or other biothiols anymore (Scheme 1b).

**Scheme 1:**
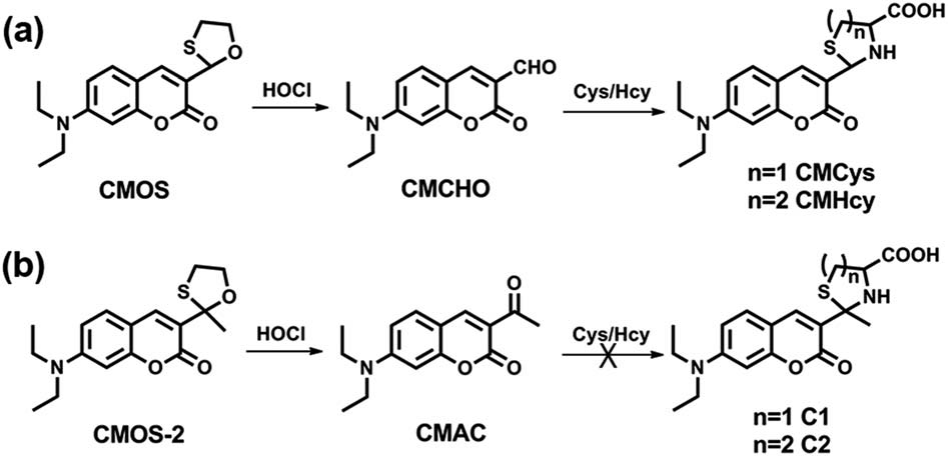
Proposed reaction mechanism of CMOS and CMOS-2 to HOCl and Cys/Hcy.

In order to confirm our design concept, the basic photo-physical characteristics of CMOS, CMCHO, CMOS-2 and CMAC were tested (Table S1, Fig. S1†). Under the excitation wavelength 405 nm, CMOS and CMOS-2 exhibited strong fluorescence centred at 480 nm in PBS buffer solution, while the fluorescence of CMCHO and CMAC was weak around this band. The emission properties of CMOS and CMCHO were also investigated at the excitation wavelength 448 nm under the same experimental conditions as well (Fig. S2†). After careful consideration, we chose 405 nm as the excitation wavelength in the follow-up experiments *in vitro* and *in vivo*.

Next, the sensitivity of CMOS and CMOS-2 to HOCl and Cys/Hcy were investigated. As we expected, both the CMOS and CMOS-2 exhibited good response to HOCl. The fluorescence intensity of CMOS and CMOS-2 decreased gradually with addition of NaOCl (Fig. 1a, S3a†), indicating that the fluorescence was switched off obviously in the presence of HOCl. The variation of intensity displayed good linearity with concentration of HOCl in the range of 0–20 μM (*R*^2^ = 0.993, Fig. S4†), and the detection limit of CMOS to HOCl was calculated to be 21 nM (S/N = 3). Subsequently, when Cys/Hcy was added to the final solution in Fig. 1a, the fluorescence intensity increased gradually within 180 min (Fig. 1b, S5†). However, the fluorescence cannot be recovered by addition thiols to the CMOS-2 solution with excess HOCl (Fig. S3b†). These results indicate that the probe CMOS can response to HOCl and Cys/Hcy in a fluorescence on-off-on manner, and can be used for monitoring the redox process with high sensitivity.

**Fig. 1:**
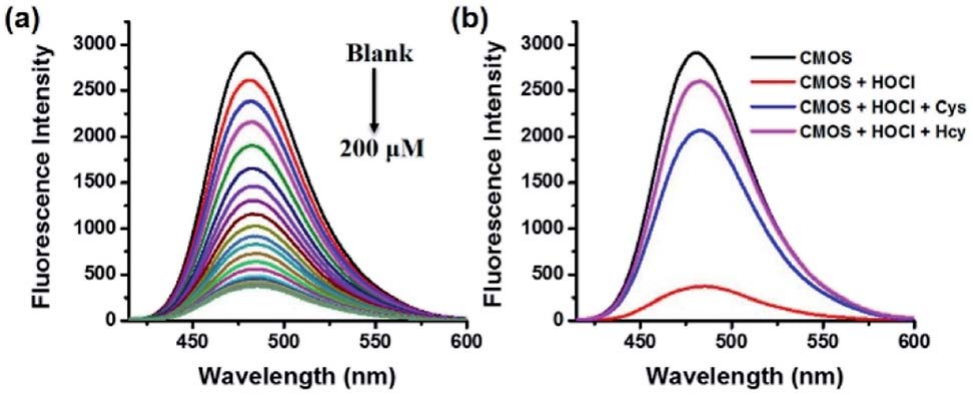
(a) Fluorescence responses of CMOS (2 μM) to different concentrations of NaOCl (0–200 μM). (b) Fluorescence responses of the CMOS solution (2 μM) with HOCl (200 μM) to Cys/Hcy (5 mM). (20 mM PBS buffer/CH_3_CN, 7 : 3, v/v, pH = 7.4, *λ*_ex_ = 405 nm).

To further identify the recognizing mechanism of probe CMOS, high performance liquid chromatography (HPLC) and mass spectral (MS) analysis were used to detect the redox process. Initially, probe CMOS displayed a single peak with a retention time at 3.7 min (Fig. 2a, S6†) while reference compound CMCHO produced a single peak with a retention time at 2.5 min (Fig. 2b, S7†). Upon the addition of HOCl to the solution of CMOS, the peak at 3.7 min weakened while 2.5 min and 2.2 min appeared (Fig. 2c). According to corresponding mass spectra, the new main peak at 2.5 min is related to compound CMCHO (Fig. S8†). The other new peak of 2.2 min corresponds to the compound C3, which can be predicted as an intermediate in the oxidation process (Fig. S8†).^28^ The addition of Cys to the solution of CMCHO also caused a new peak with a retention time at 2.1 min, which has been confirmed to be the thioacetal product CMCys (Fig. S9†). The possible sensing mechanism is depicted in Fig. S10.†

**Fig. 2:**
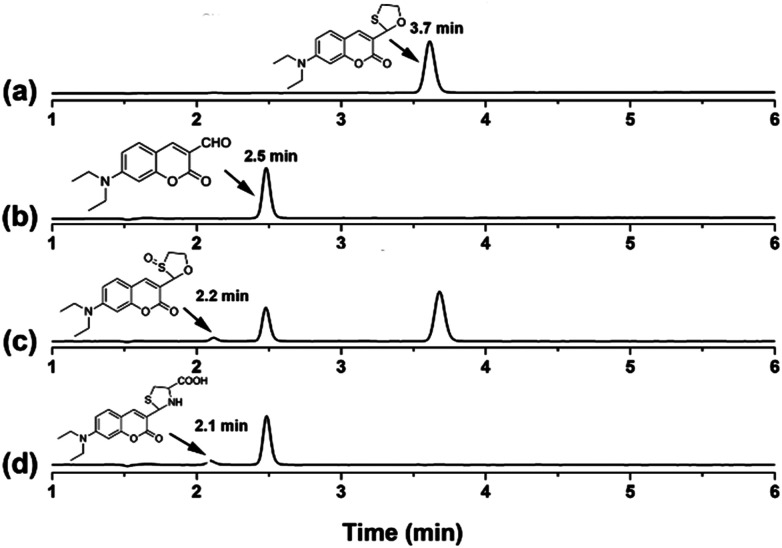
The reversed-phase HPLC with absorption (400 nm) detection. (a) 10 μM CMOS. (b) 10 μM CMCHO. (c) 10 μM CMOS in the presence of 50 μM HOCl for 30 s. (d) 10 μM CMCHO in the presence of 1 mM Cys for 30 min. (Eluent: CH_3_CN containing 0.5% CH_3_COOH; 100% CH_3_CN (0–7 min), 0.5 ml min^−1^, 25 °C; injection volume, 5.0 μL).

To study the selectivity of CMOS towards HOCl, we performed fluorescence response to different reactive oxygen species (ROS), reactive nitrogen species (RNS) and reactive sulfur species (RSS). As shown in Fig. 3a, CMOS exhibited significant change of fluorescence intensity only in the presence of HOCl, while other ROS and RNS, such as singlet oxygen (^1^O_2_), hydrogen peroxide (H_2_O_2_), hydroxyl radical (HO·), superoxide anion (O_2_^−^), nitric oxide (NO), *tert*-butylhydroperoxide (*t*-BuOOH) and *tert*-butoxy radical (*t*-BuOO·) had no obvious fluorescence emission changes. Additionally, RSS which are abundant in biological samples, showed no influence in this process under the identical condition. The detection of reducing process was also investigated. As displayed in Fig. 3b, only cysteine and homocysteine induced excellent fluorescence recovery towards other reducing materials, such as RSS and various amino acids. Furthermore, the selectivity of CMOS-2 was also studied in the same condition. As expected, CMOS-2 could selectively detect HOCl, and not alter fluorescence intensity under various kinds of biothiols (Fig. S11†). Therefore, our design strategy for the on–off–on probe is confirmed by results obtained above, with which CMOS can be utilized for detecting the redox process between HOCl and Cys/Hcy with high selectivity.

**Fig. 3:**
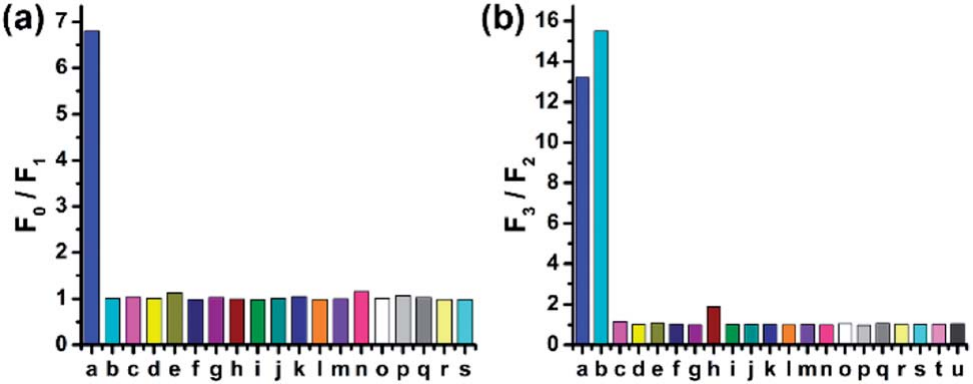
(a) Fluorescence response of CMOS (2 μM) to different ROS, RNS and RSS (200 μM). Bars represent emission intensity ratios before (*F*_0_) and after (*F*_1_) addition of each analytes. (a) HOCl; (b) KO_2_; (c) H_2_O_2_; (d) ^1^O_2_; (e) HO·; (f) *t*-BuOOH; (g) *t*-BuOO·; (h) NO_2_^−^; (i) NO_3_^−^; (j) NO; (k) GSH; (l) Cys; (m) Hcy; (n) Na_2_S; (o) Na_2_S_2_O_3_; (p) Na_2_S_2_O_8_; (q) NaSCN; (r) DTT; (s) Na_2_SO_3_. (b) Fluorescence response of the solution added HOCl in (a) to different RSS and amino acids. Bars represent emission intensity ratios before (*F*_2_) and after (*F*_3_) addition of each analytes (5 mM). (a) Cys; (b) Hcy; (c) Na_2_S; (d) Na_2_S_2_O_3_; (e) Na_2_S_2_O_8_; (f) NaSCN; (g) DTT; (h) Na_2_SO_3_; (i) Ala; (j) Glu; (k) Gly; (l) His; (m) Ile; (n) Leu; (o) Met; (p) Phe; (q) Pro; (r) Ser; (s) Trp; (t) Vc; (u) GSH. (20 mM PBS buffer/CH_3_CN, 7 : 3, v/v, pH = 7.4, *λ*_ex_/*λ*_em_ = 405/480 nm).

Subsequently, the influence of pH on probe CMOS was measured. The fluorescence intensity of CMOS and CMCHO perform no significant variances in wide pH ranges (pH = 4–11, Fig. S12a†). Fluorescence intensity changes could be observed immediately when HOCl was added into the solution of probe CMOS, especially in alkaline condition (Fig. 4a). Considering the p*K*_a_ of HOCl is 7.6,^29^CMOS is responsive to both HOCl and OCl^−^. Alkaline condition was also benefit for the fluorescence recovery of CMOS from Cys/Hcy (Fig. S12b†). It is reasonable to consider that thiol atom displays higher nucleophilicity in alkaline condition. From the stop-flow test, the UV-visible absorbance of probe CMOS sharply decreased at the wavelength of 400 nm (Fig. 4b). The response time was within 10 s and the kinetic of the reaction was fitted to a single exponential function (*k*_obs_ = 0.67 s^−1^). The ability of instantaneous response is extremely necessary to intracellular HOCl detection.

**Fig. 4:**
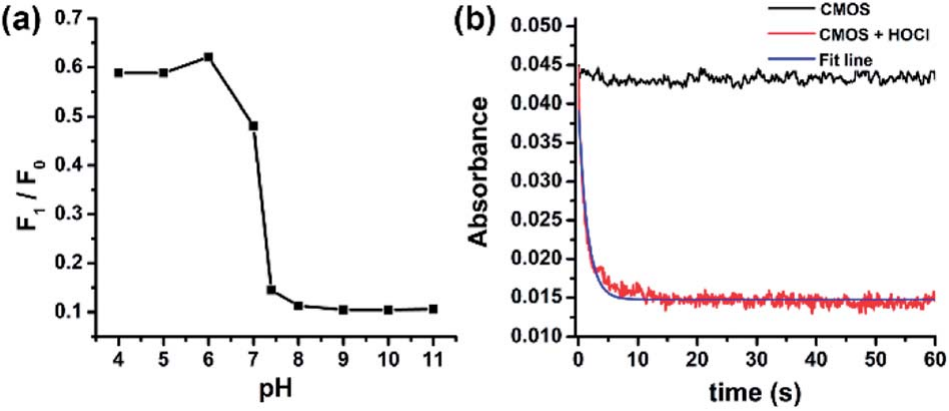
(a) Fluorescence responses of CMOS to HOCl under different pH values. Squares represent emission intensity ratios after (*F*_1_) and before (*F*_0_) addition of 200 μM HOCl (*λ*_ex_/*λ*_em_ = 405/480 nm). (b) Time-dependent changes in the absorption intensity of CMOS (1 μM) before and after addition of HOCl. (20 mM PBS buffer/CH_3_CN, 7 : 3, v/v, pH = 7.4, *λ*_abs_ = 400 nm).

With these data in hand, we next applied CMOS for fluorescence imaging of the redox changes with HOCl and Cys/Hcy in living cells. After incubation with 5 μM CMOS at 37 °C for 30 min, intense fluorescence was observed of the SKVO-3 cells in the optical window 425–525 nm (Fig. 5a and d), indicating the probe can easily penetrate into cells. Treating the cells with 100 μM NaOCl led to remarkable fluorescence quenching as the probe sensed the HOCl-induced oxidative stress (Fig. 5b and e). After 3 min, the cells were washed with PBS buffer three times, and added 5 mM Cys/Hcy for 1 h, respectively. Then the fluorescence was recovered obviously (Fig. 5c and f). Experimental results clearly declare that the probe CMOS was successfully used to detect the process of HOCl oxidative stress and Cys/Hcy reducing repair in living cells.

**Fig. 5:**
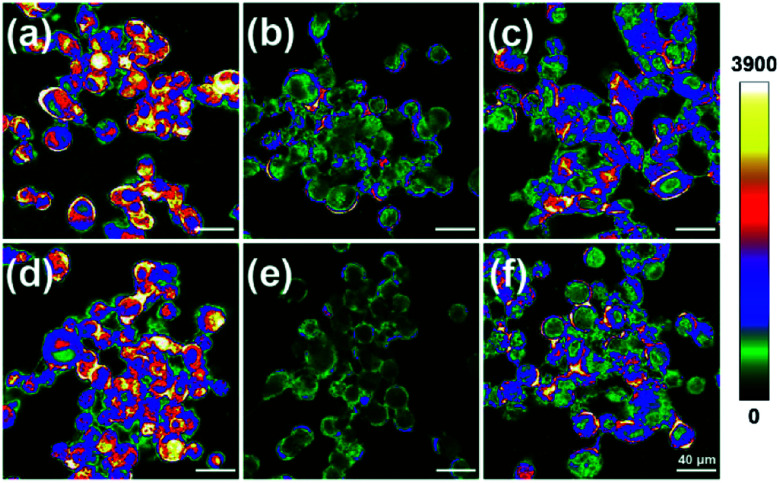
Fluorescence imaging of the process of HOCl oxidative stress and thiols repair in CMOS-labeled SKVO-3 cells. Fluorescence images of SKVO-3 cells loaded with 5 μM CMOS at 37 °C for 30 min (a and d). Dye-loaded cells treated with 100 μM NaOCl at 25 °C for 3 min (b and e). Dye-loaded, NaOCl-treated cell incubated with 5 mM Cys (c), 5 mM Hcy (f) for 1 h. Emission intensities were collected in an optical window 425–525 nm, *λ*_ex_ = 405 nm, intensity bar: 0–3900.

## Conclusions

In this work, a novel on–off–on fluorescent probe was reported for highly selective detection HOCl oxidative stress and Cys/Hcy reducing repair *in vivo* and *in vitro*. The probe CMOS can be easily synthesized and displayed high sensitivity, fast response, and high selectivity. Cells images indicated that CMOS was capable to sense the redox changes between HOCl and Cys/Hcy. Results show that the probe CMOS would be a potential tool to study the oxidative damage and biothiols repairs in the biology and medical research.

## Conflicts of interest

There are no conflicts to declare.

## Supplementary Material

RA-008-C7RA13419C-s001
