# Supplementary material for: A novel fluorescent probe for imaging the process of HOCl oxidation and Cys/Hcy reduction in living cells
Source: RSC Adv. 2018 Mar 5;8(17):9519–23. doi: 10.1039/c7ra13419c (PMC9078674; doi:10.1039/c7ra13419c)
Supplement: RA-008-C7RA13419C-s001 [file RA-008-C7RA13419C-s001.pdf]

Electronic Supplementary Material (ESI) for RSC Advances.
This journal is © The Royal Society of Chemistry 2018

Electronic Supplementary Information

A Novel Fluorescent Probe for Imaging the Process of HOCl Oxidation and Cys/Hcy Reduction in Living Cells

Zhen Liu,[a] Guoping Li, *[a] Yana Wang, [a] Jiulong Li,[a] Yang Mi,[b] Linna Guo,[a] Wenjian Xu,[a] Dapeng Zou,[a] Tiesheng Li *[a] and Yangjie Wu *[a]

[a] The College of Chemistry and Molecular Engineering, Zhengzhou University, Zhengzhou 450001, Henan Province, P. R. China.
[b] School of Basic Medical Sciences, Zhengzhou University, Zhengzhou 450001, Henan Province, P. R. China.

1. General Experimental Procedures S2
2. Synthesis and Characterization of Compounds S3
3. Spectra of UV-visible Absorbance and Fluorescence, and HPLC-MS Analysis S4
4. NMR data S9
5. HR-MS data S11
6. References S12

1. General Experimental Procedures

Reagents and Apparatus.

Methanesulfonic acid, 4-(diethylamino)-salicyladehyde, 2-mercaptoethanol, dimethyl sulfoxide (DMSO), sodium hypochlorite (NaOCl, 14.5% available chlorine), *tert*-butylhydroperoxide (*t*-BuOOH, 70%) were purchased from Aladdin and used as received. UV/Vis spectra were recorded on a GBC Cintra 2020 UV-vis spectrometer. Fluorescent spectra measurements were obtained on a HITACHI F-4500 fluorescent spectrophotometer. NMR spectra were performed on a Bruker DPX-400 NMR spectrometer. HPLC-MS were obtained on Agilent 1100 series and LC/MSD Trap XCT. High resolution mass spectra were ensured on a MALDI-FTMS. The stop-flow test was obtained on an Applied Photophysics Chirascan Series spectrometers. The path length was 1 cm with cell volume of 3.0 mL. All the spectroscopic measurements in this supporting information were carried out in PBS buffer (20 mM PBS buffer/CH_3CN, 7:3, v/v, pH = 7.4, λ_{ex} = 405 nm) with stirring at room temperature. Confocal fluorescence imaging experiments were performed with an Olympus FV-1000 laser scanning microscopy system, based on an IX81 (Olympus, Japan) inverted microscope. Images were collected and processed with Olympus FV10-ASW Ver.2.1b software.

Preparation of ROS and RNS

Hypochlorous acid (HOCl), hydrogen peroxide (H_2O_2) and *tert*-butylhydroperoxide (*t*-BuOOH) stock solutions were prepared by dilution of commercial NaOCl solution (14.5% available chlorine), H_2O_2 (30%) and *t*-BuOOH (70%) in deionized water. Superoxide anion (O_2^-), Hydroxyl radical (HO•) and Singlet oxygen ($^1O_2$) were prepared as previous report [S1]: Superoxide anion (O_2^-) was prepared from KO_2 (2 mg) in dry DMSO (2 mL) with vigorously stirring; [S2] Hydroxyl radical (HO•) and *t*-BuOO• was generated in situ by Fenton reaction from 200 μM H_2O_2 or *t*-BuOOH and $FeSO_4$ (1 mM); [S3] Singlet oxygen ($^1O_2$) was generated in situ by the H_2O_2/MoO_4^{2-} (200 μM/1 mM) system in alkaline media. [S4] Nitrate and nitrite stock solutions were prepared by commercial $NaNO_3$ and $NaNO_2$ solid in deionized water. $ONOO^-$ was generated from $NaNO_2$ and H_2O_2, and quantified according to literature. [S5] Nitric oxide (NO) was generated from SNP (Sodium Nitroferricyanide (III) Dihydrate).

Methods of Cell Culture

Human epithelial ovarian cancer cell SKOV-3 cells were cultured in Dulbecco's modified Eagle's medium (DMEM, Gibco) supplemented with 10% fetal calf serum (FCS, Gibco), 50 μg mL^{-1} penicillin/streptomycin (Hyclone) at 37 °C in a humidified incubator containing 5% CO_2 gas. The cells were plated in a 35 mm glass-bottomed dish and cultured for 2 days before dye loading. Then the cells were washed with phosphate-buffered saline (PBS) and bathed in corresponding serum-free DMEM/RPMI-1640 medium with 5 μM **CMOS** for 20 min at 37 °C, washed with PBS three times to remove the excess probe and bathed in PBS (2 mL) before imaging.

2. Synthesis and Characterization of Compounds

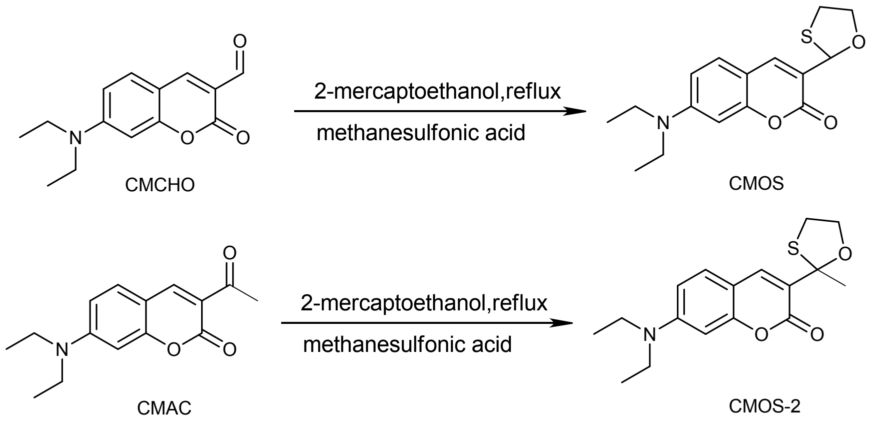

Scheme S1 Synthetic route of **CMOS** and **CMOS-2**

Preparation of CMCHO and CMCA Compound **CMCHO** and **CMCA** was synthesized according to the literature procedure. [S6]

Preparation of CMOS 7-(diethylamino)-2-oxo-2H-chromene-3-carbaldehyde (100 mg, 0.41 mmol), methanesulfonic acid (160 μL) and 2-mercaptoethanol (41.6 mg, 0.48 mmol) were mixed in 6 mL dichloromethane solution and refluxed for 3 h under nitrogen protection. Then the mixture was purified by preparative TLC with a solvent system (ethyl acetate: petroleum ether = 2:1). Light yellow solid product was obtained.

CMOS. Yield: 44.3 mg (35.6%). ^{1}H NMR (400 MHz, $CDCl_3$) δ 7.78 (1H, s), 7.29 (1H, d, J = 8.8Hz), 6.62 (1H, d, J = 8.7Hz), 6.52 (1H, s), 6.13 (1H, s), 4.52-4.48 (1H, m), 4.04-3.98 (1H, m), 3.44 (4H, q, J = 7.1Hz), 3.16-3.12 (2H, m), 1.22 (6H, t, J = 7.1Hz). ^{13}C NMR (100 MHz, $CDCl_3$) δ 161.13, 155.82, 150.51, 138.13, 129.09, 119.90, 108.87, 107.97, 96.89, 71.98, 44.65, 32.82,12.29. HR-MS $C_{16}H_{20}NO_3S^+$ [M+H$^+$], found 306.1168, calculated 306.1158.

Preparation of CMOS-2 3-acetyl-7-(diethylamino)-2H-chromen-2-one (100 mg, 0.38 mmol), methanesulfonic acid (140 μL) and 2-mercaptoethanol (34.7 mg, 0.40 mmol) were mixed in 6 mL dichloromethane solution and refluxed for 3 h under nitrogen protection. Then the mixture was purified by preparative TLC with a solvent system (ethyl acetate: petroleum ether = 2: 1). Yellow solid product was obtained.

CMOS-2. Yield: 73.9 mg (60.1%). ^{1}H NMR (400 MHz, $CDCl_3$) δ 7.63 (1H, s), 7.28 (1H, d, J = 8.8Hz), 6.61 (1H, d, J = 8.4Hz), 6.52 (1H, d, J = 1.8Hz), 4.42-4.37 (1H, m), 4.17-4.13 (1H, m), 3.43 (4H, q, J = 7.1Hz), 3.17-3.11 (1H, m), 2.99-2.94 (1H, m), 1.94 (3H, s), 1.21 (6H, t, J = 7.1Hz). ^{13}C NMR (100 MHz, $CDCl_3$) δ 160.81, 155.72, 150.11, 134.56, 128.92, 125.64, 108.78, 107.94, 97.02, 91.73, 71.04, 44.68, 33.21, 29.44, 12.24. HR-MS $C_{17}H_{22}NO_3S^+$ [M+H$^+$], found 320.1323, calculated 320.1315.

3. Spectra of UV-visible Absorbance and Fluorescence, and HPLC-MS Analysis

Table S1 Photophysical parameters of fluorophores

Dye	λ_{max}(nm) [a]	ε_{max}(10^4 cm^{-1} mol^{-1})[b]	λ_{em}(nm) [c]	Φ [d]
CMOS	398	2.62	480	0.10
CMCHO	451	4.92	491	0.01
CMOS-2	394	2.87	475	0.24
CMAC	448	6.77	490	0.01

[a] Maximum absorbance wavelength of fluorophores in PBS buffer (20 mM PBS buffer/CH_3CN, 7:3, v/v, pH = 7.4, λ_{ex} = 405 nm). [b] The molar extinction coefficients at maximum wavelength .[c] Maximum fluorescence emission wavelength in PBS buffer (20 mM PBS buffer/CH_3CN, 7:3, v/v, pH = 7.4, λ_{ex} = 405 nm). [d] The relative fluorescence quantum yields were measured in PBS solution using quinine-sulfate (Φ = 0.54 in 0.1 M H_2SO_4) as a reference.

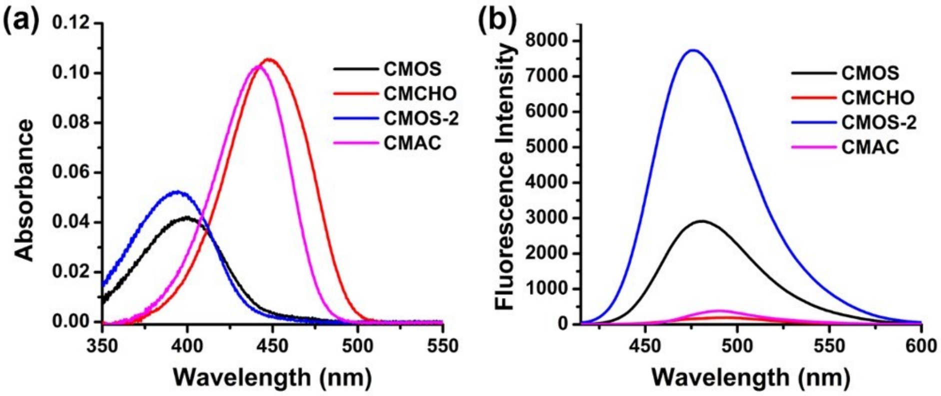

Fig. S1 (a) UV-visible Absorbance spectra and (b) Fluorescence spectra of 2 μM **CMOS**, **CMCHO**, **CMOS-2**, and **CMAC**. (20 mM PBS buffer/CH_3CN, 7:3, v/v, pH = 7.4, λ_{ex} = 405 nm).

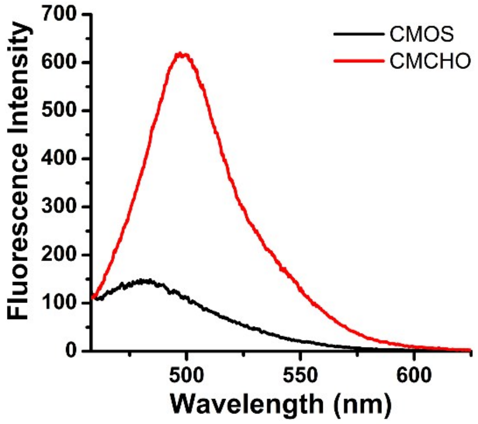

Fig. S2 Fluorescence spectra of 2 μM **CMOS** and **CMCHO**. (20 mM PBS buffer/CH_3CN, 7:3, v/v, pH = 7.4, λ_{ex} = 448 nm).

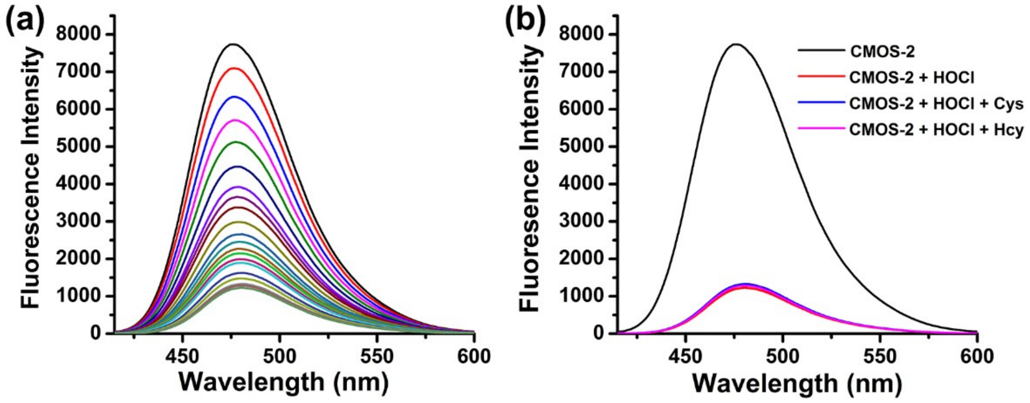

Fig. S3 (a) Fluorescence responses of **CMOS-2** (2 μM) to different concentrations of HOCl (0-200 μM). (b) Fluorescence responses of the **CMOS-2** solution (2 μM) with HOCl (200 μM) to Cys/Hcy (5 mM). (20 mM PBS buffer/CH_3CN, 7:3, v/v, pH = 7.4, λ_{ex} = 405 nm).

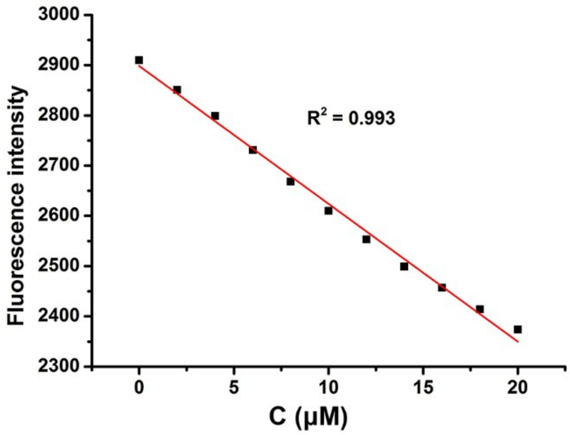

Fig. S4 Fluorescence intensities of 2 μM **CMOS** at 480 nm as a function of the concentrations of HOCl in the range of 0-20 μM (20 mM PBS, PBS/CH_3CN = 7:3 v/v, pH = 7.4, λ_{ex} = 405 nm).

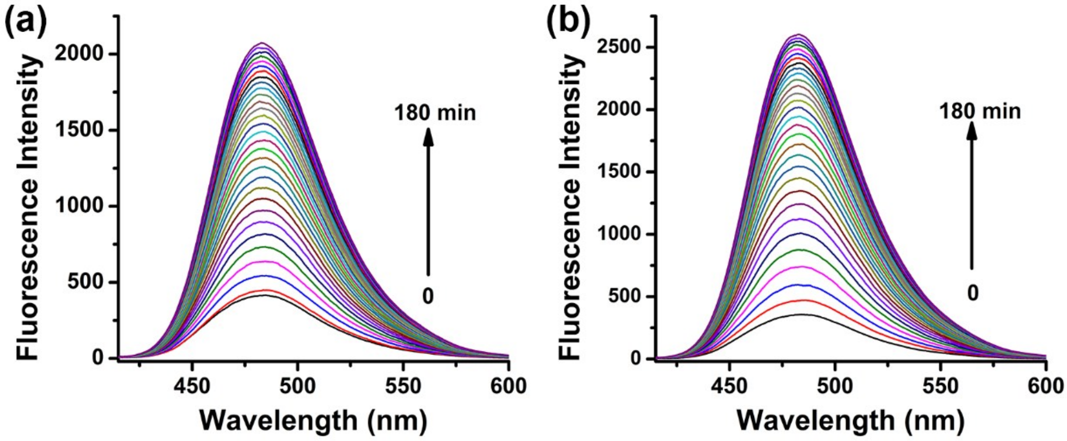

Fig. S5 Fluorescence intensity enhancement of **CMOS** (2 μM) added excess HOCl and 5 mM Cys (a) or Hcy (b) in 180 mins (20 mM PBS, PBS/CH_3CN = 7:3 v/v, pH = 7.4, λ_{ex} = 405 nm).

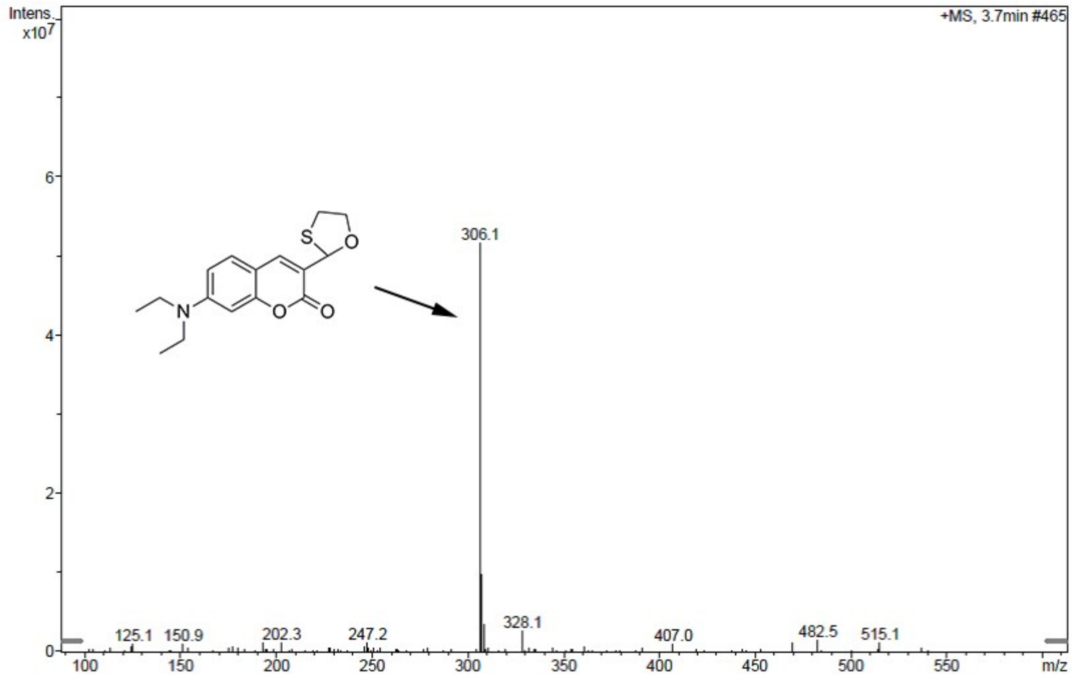

Fig. S6 The mass spectrum corresponding to Fig. 2a.

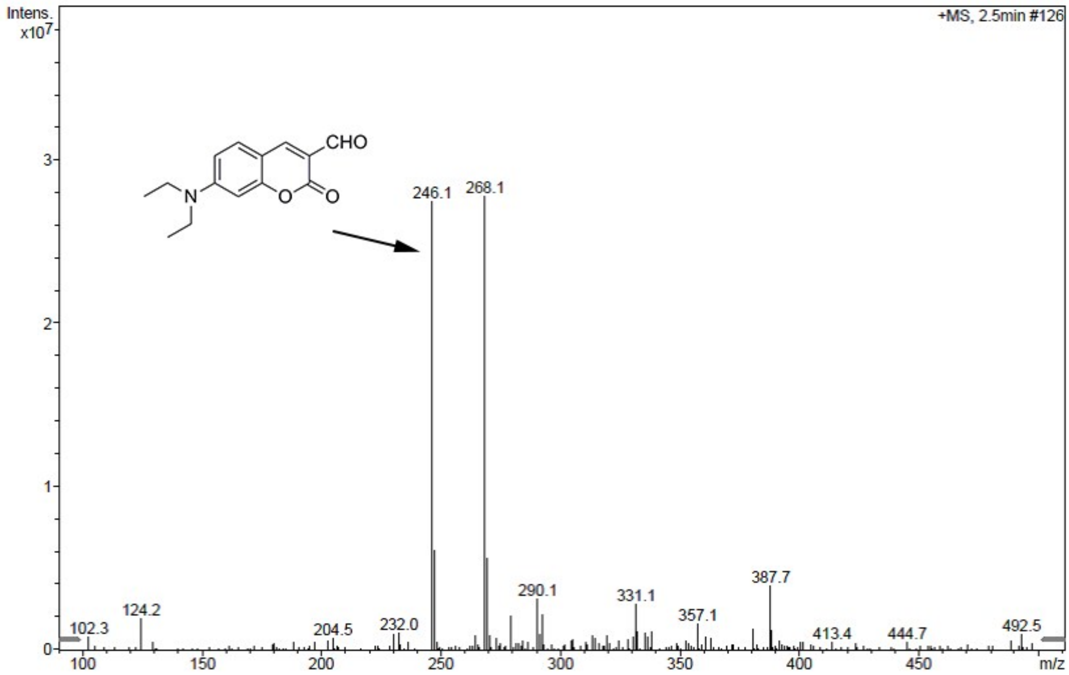

Fig. S7 The mass spectrum corresponding to Fig. 2b.

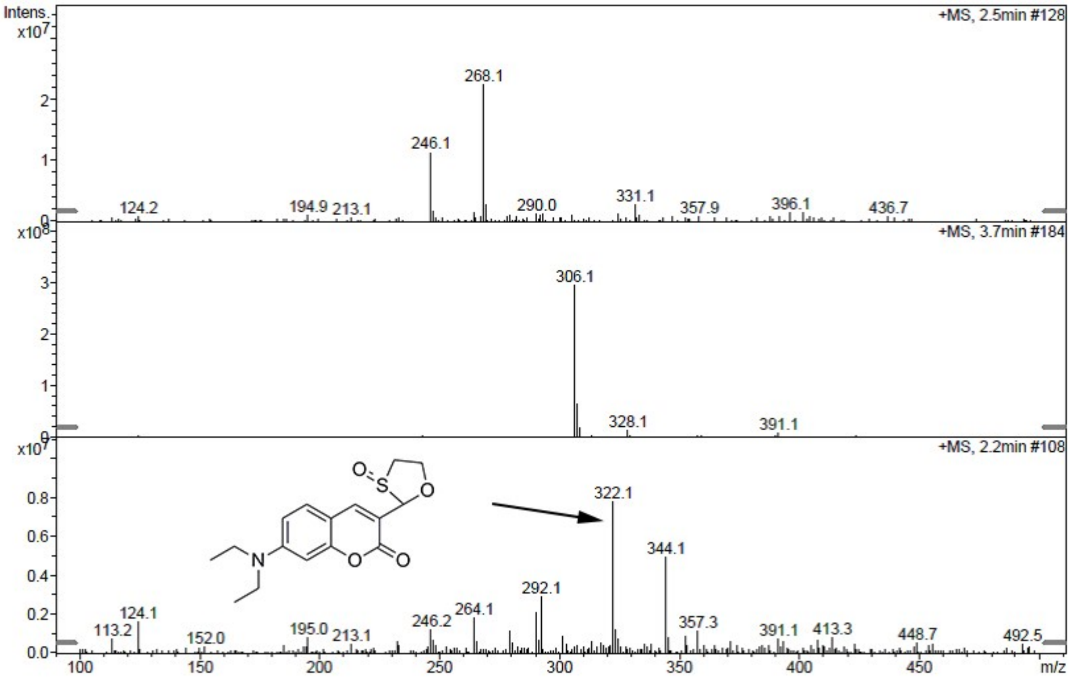

Fig. S8 The mass spectra corresponding to Fig. 2c.

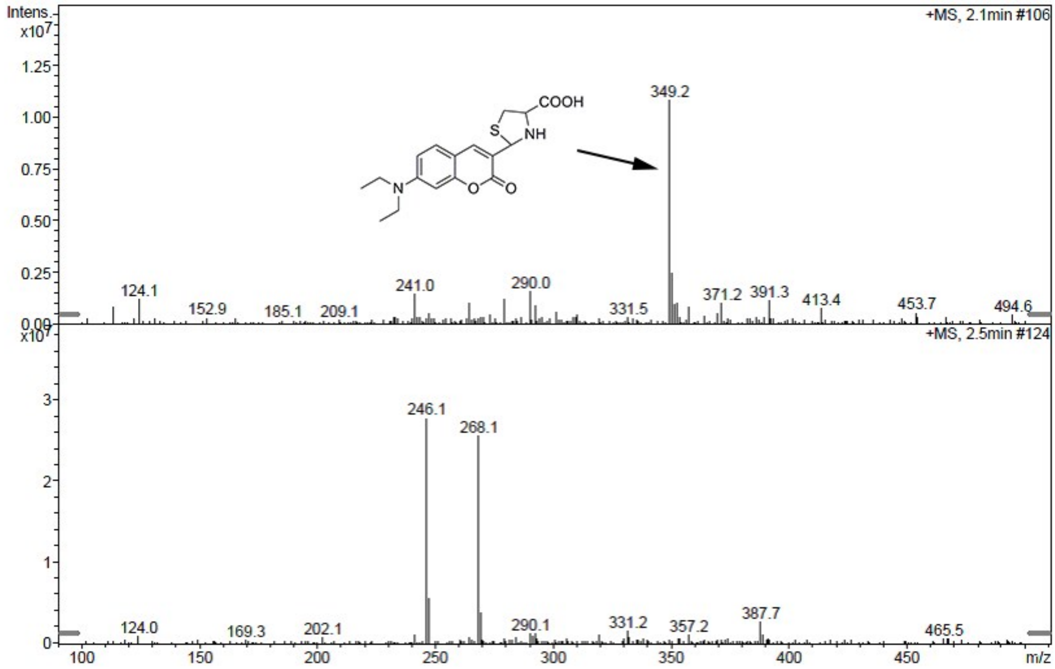

Fig. S9 The mass spectra corresponding to Fig. 2d.

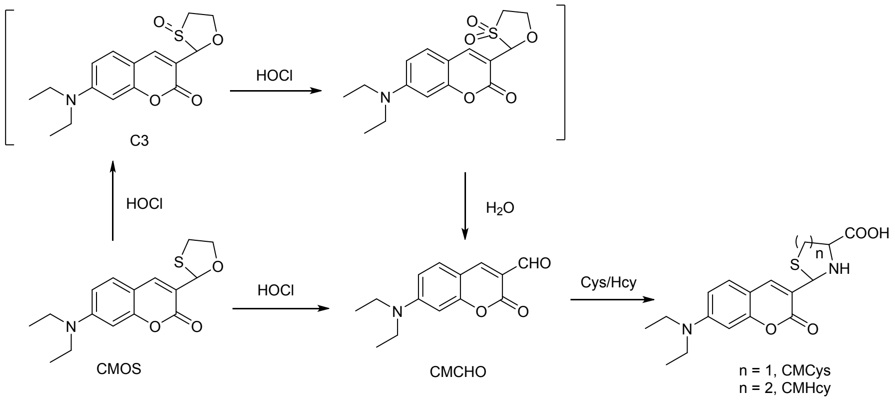

Fig. S10 Proposed sensing mechanism of **CMOS** responding to HOCl and Cys/Hcy.

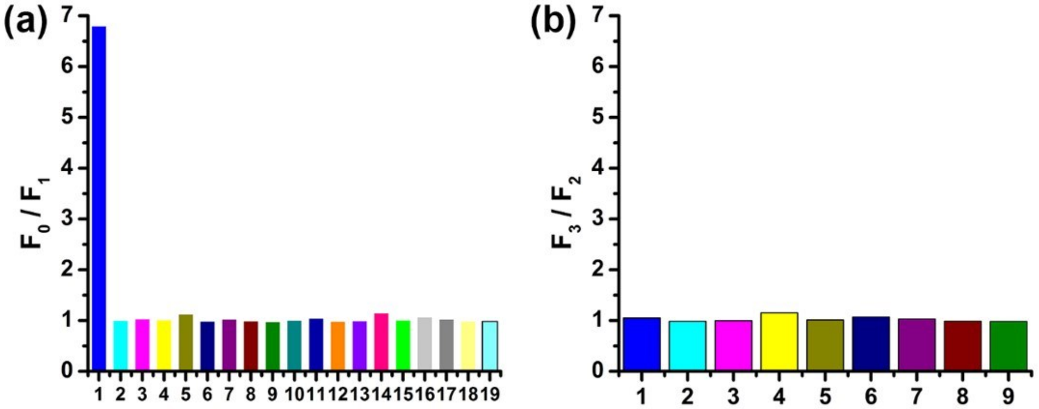

Fig. S11 (a) Fluorescence response of 2 μM **CMOS-2** to different ROS, RNS and RSS. Bars represent emission intensity ratios before (F_0) and after (F_1) addition of each analytes (200 μM). 1: HOCl; 2: KO_2; 3: H_2O_2; 4: $^1O_2$; 5: HO·; 6: *t*-BuOOH; 7: *t*-BuOO·; 8: NO_2^-; 9: NO_3^-; 10: NO; 11: GSH; 12: Cys; 13: Hcy; 14: Na_2S; 15: $Na_2S_2O_3$; 16: $Na_2S_2O_8$; 17: NaSCN; 18: DTT; 19: Na_2SO_3. (b) Fluorescence response of of the solution added NaOCl in Fig. S10a to different RSS and amino acids. Bars represent emission intensity ratios before (F_2) and after (F_3) addition of each analytes (5 mM). 1: Cys; 2: Hcy; 3: Na_2S; 4: $Na_2S_2O_3$; 5: $Na_2S_2O_8$; 6: NaSCN; 7: DTT; 8: Na_2SO_3; 9: GSH. (20 mM PBS buffer/CH_3CN, 7:3, v/v, pH = 7.4, $\lambda_{ex}/\lambda_{em}$ = 405/480 nm).

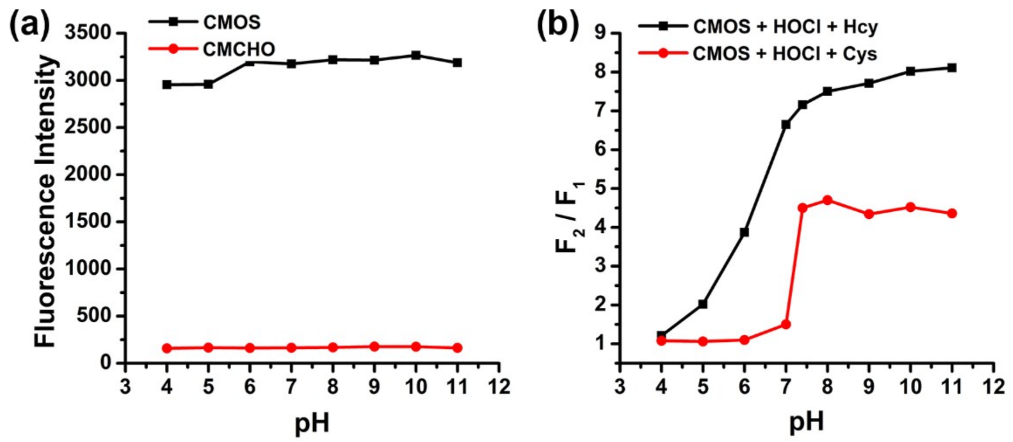

Fig. S12 (a) Fluorescence intensity of probe **CMOS** and **CMCHO** in various pH values. (b) Plot of fluorescence intensity ratio changes of **CMOS** solution added excess HOCl (F_1) and 5 mM Cys/Hcy after 2 hours (F_2) in various pH values. (20 mM PBS buffer/CH_3CN, 7:3, v/v, $\lambda_{ex}/\lambda_{em}$ = 405/480 nm).

4. NMR data

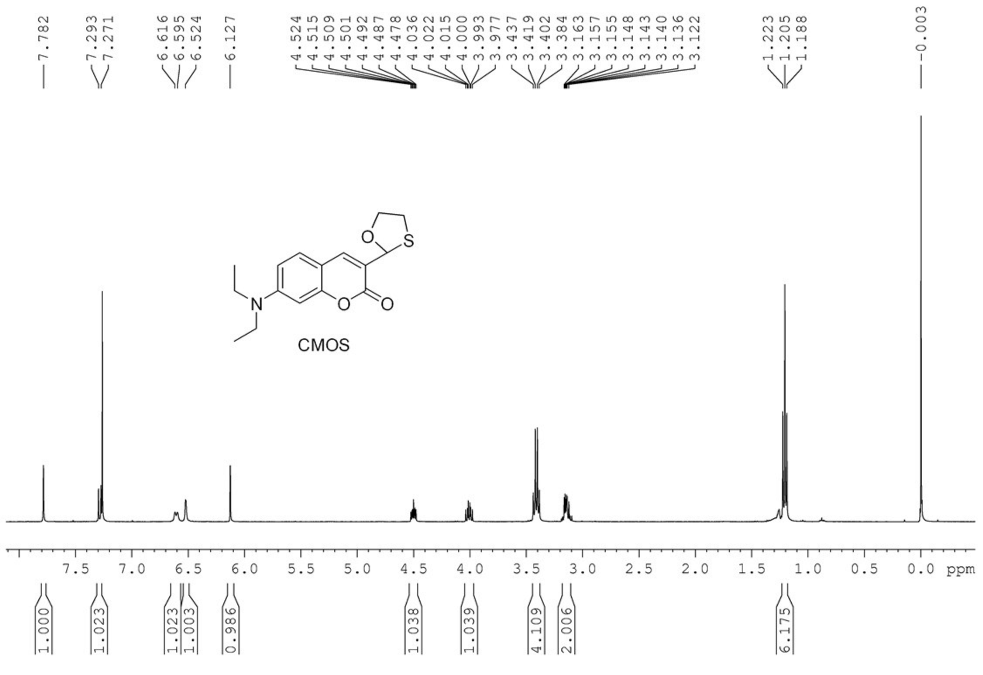

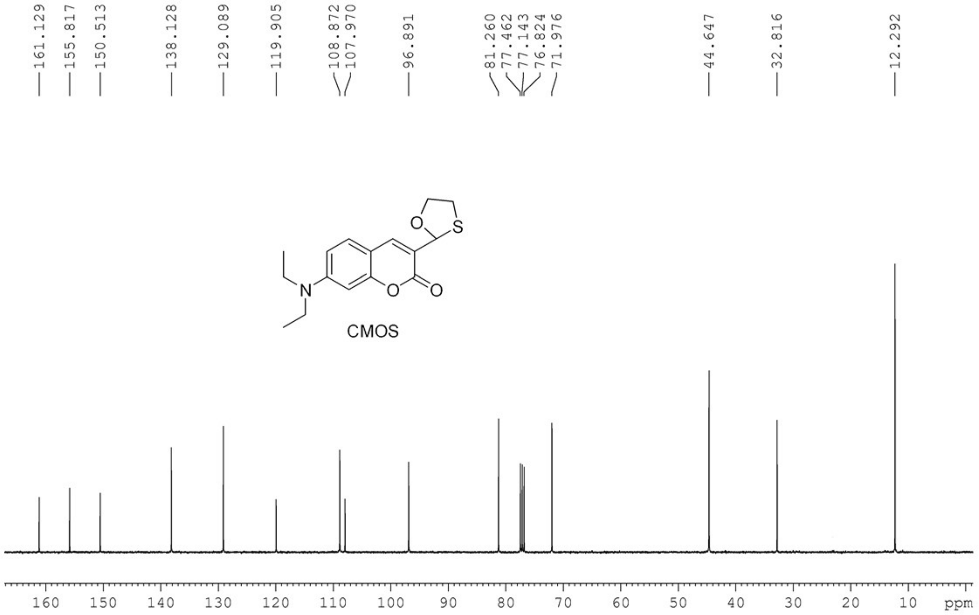
CMOS
ppm

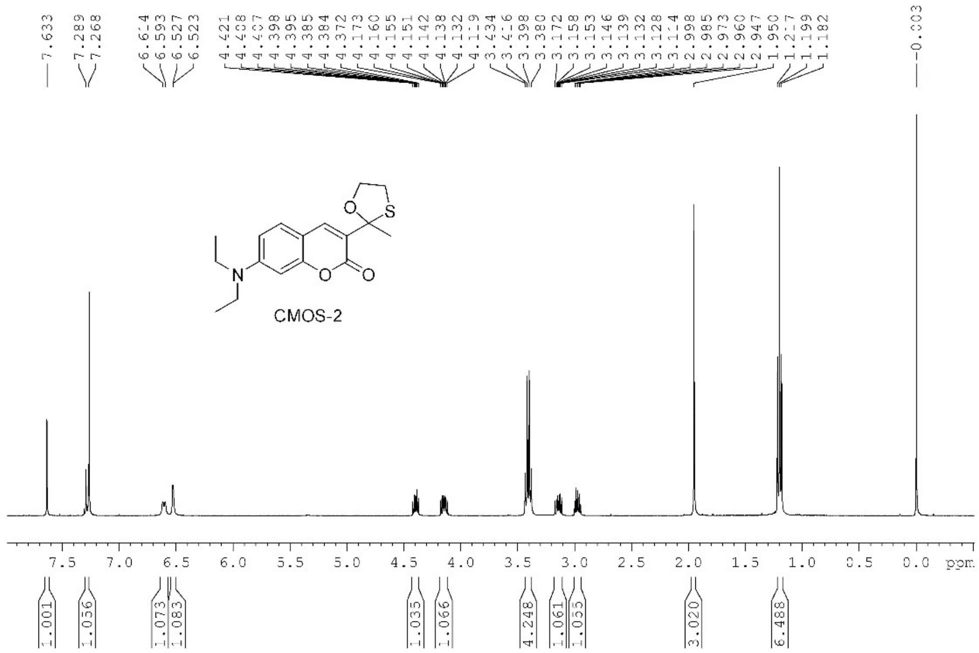
CMOS-2
ppm

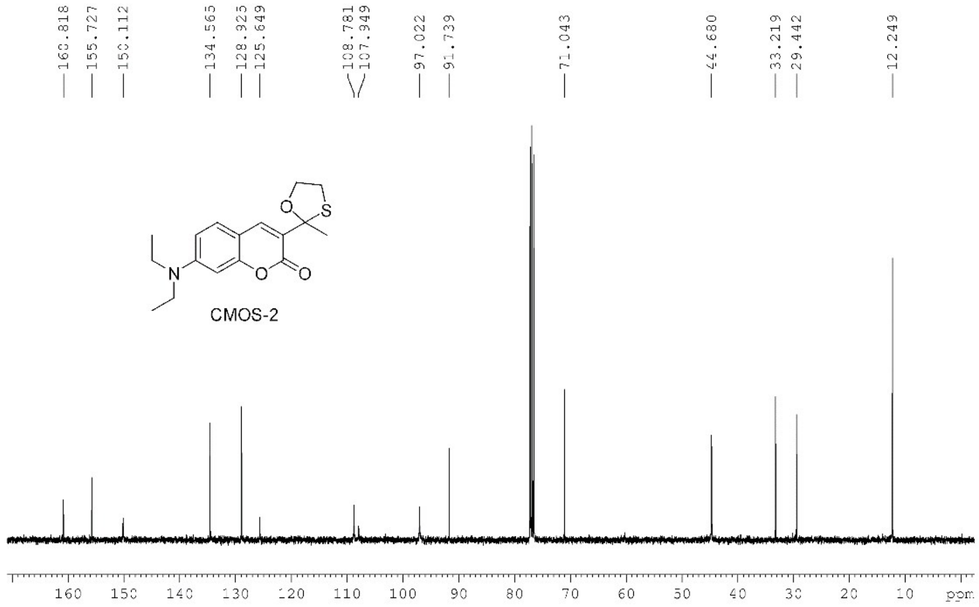

160.818
155.727
150.112
134.565
128.925
125.649
108.781
107.949
97.022
91.739
71.043
44.680
33.219
29.442
12.249
CMOS-2
160 150 140 130 120 110 100 90 80 70 60 50 40 30 20 10 ppm

5. HR-MS data

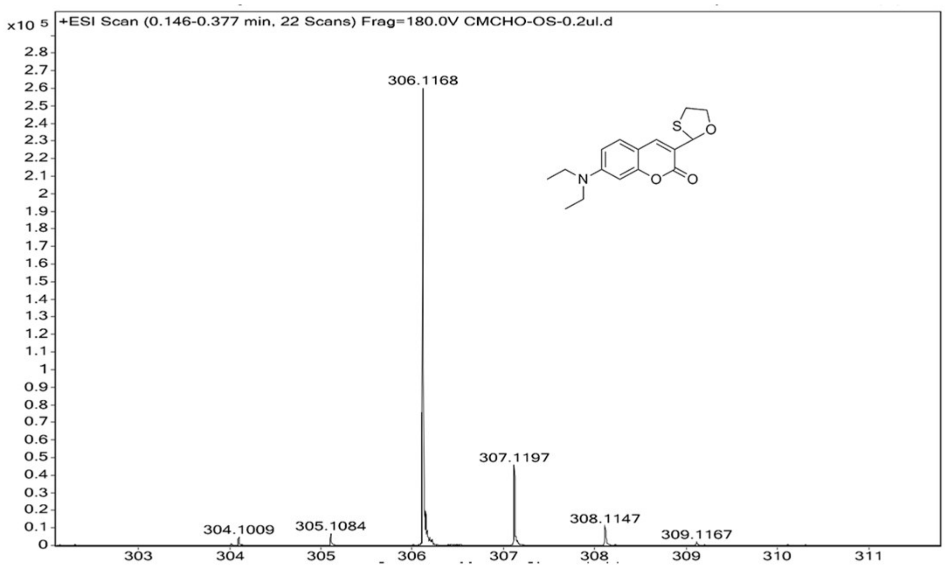

x10 5
+ESI Scan (0.146-0.377 min, 22 Scans) Frag=180.0V CMCHO-OS-0.2ul.d
306.1168
307.1197
308.1147
309.1167
304.1009
305.1084
303 304 305 306 307 308 309 310 311

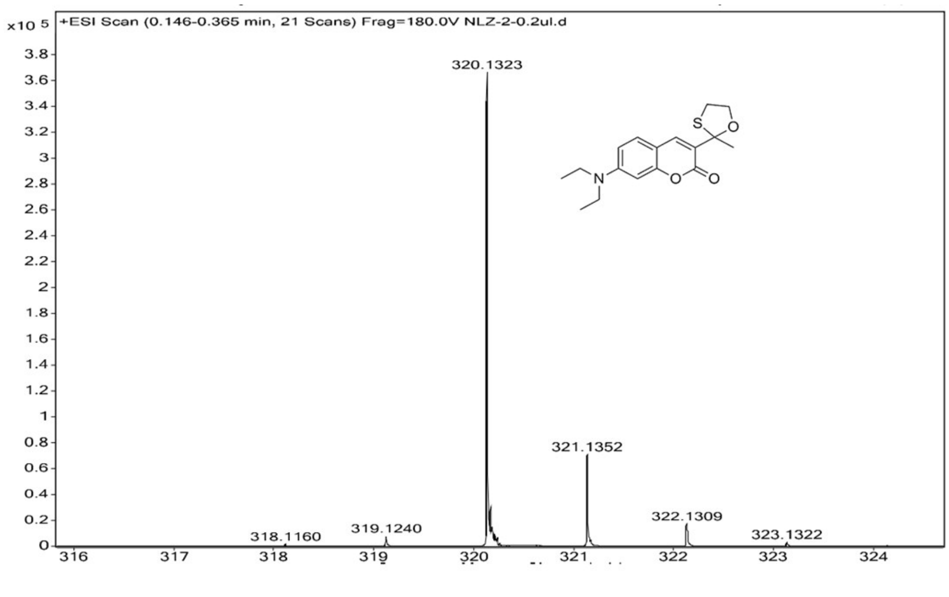

6. References

(S1) Chen, S.; Lu, J.; Sun, C.; Ma, H. *Analyst*. **2010**, *135*, 577-582.

(S2) Zhao, H. T.; Kalivendi, S.; Zhang, H.; Joseph, J.; Nithipatikom, K.; Vasquez-Vivar, J.; Kalyanaraman, B. *Free Radical Biol. Med.* **2003**, *34*, 1359-1368.

(S3) Setsukinai, K.; Urano, Y.; Kakinuma, K.; Majima, H. J.; Nagano, T. *J. Biol. Chem.* **2003**, *278*, 3170-3175.

(S4) Aubry, J. M.; Cazin, B.; Duprat, F. *J. Org. Chem.* **1989**, *54*, 726-728.

(S5) Nakagawa, H.; Sumiki, E.; Ikote, N.; Matsushima, Y.; Ozawa, T. *Antioxid. Redox. Signal.* **1999**, *1*, 239-244.

(S6) Wu, J, S.; Sheng, R, L.; Liu, W, M.; Wang, P, F.; Zhang, H, Y.; Ma, J, J. *Tetrahedron* **2012**. *68*, 5458-5463.
